# Palliative sedation rate for noncancer terminally ill patients at home in Japan

**DOI:** 10.1186/s12904-025-01799-y

**Published:** 2025-06-04

**Authors:** Moyuru Shionozaki, Ryo Yamamoto, Jun Hamano

**Affiliations:** 1 Department of General Practice, Saku Central Hospital, 197 Usuda, Saku, Nagano, Japan; 2 Department of Palliative Medicine, Saku Central Hospital Advanced Care Center, 3400-28 Nakagomi, Saku, Nagano, Japan; 3 Department of Palliative and Supportive Care, Institute of Medicine, University of Tsukuba, 1-1-1 Tennoudai, Tsukuba, Ibaraki 305-8575 Japan

**Keywords:** Palliative sedation, Noncancer patients, Home care

## Abstract

**Background:**

Previous studies have investigated the frequency and target symptoms of palliative sedation in patients with home-based cancer. However, the status of home-based non-cancer patients remains unclear. This study aimed to determine the frequency and target symptoms of palliative sedation in home-based non-cancer patients in Japan.

**Methods:**

We conducted a post hoc analysis of a multicenter prospective cohort study of elderly non-cancer patients at home in Japan between January 2020 and December 2020. The physicians routinely assessed and recorded symptoms and treatment every 3 months until home care was discontinued or until the patient died at home. This multicenter prospective cohort study targeted non-cancer patients aged 65 years and over receiving care at home or in nursing homes.

**Results:**

Of the 785 patients, 195 died at home or in nursing homes. Seven patients (3.6%) received palliative sedation before death. The target symptoms for sedation were delirium and dyspnea.

**Conclusions:**

The sedation rate for noncancer patients at home is relatively low, and the major target symptoms of sedation are delirium and dyspnea.

## Background

Dissemination and awareness of palliative care for noncancer patients is important [1]. Noncancer older people with multiple comorbidities suffer more frequently and significantly [2]. If the refractory symptoms cannot be resolved with medication or other alternatives, sedation is necessary. There are reports of patients with cancer on the frequency of palliative sedation or details of sedation in palliative care wards or home care settings [3–5]. However, there is a lack of data on palliative sedation for non-cancer patients in the home setting, and international discussions on this topic remain inadequate. Hence, we aimed to explore the details of palliative sedation in home care.

## Method

This was a prospective cross-sectional observational study. This study was a secondary data analysis of a survey conducted as part of the Epochal-J study. The details of this study have been previously published [6]. The research plan states that data from the primary study may be used in a secondary manner that is not linked to personally identifiable information. The Epochal-J study explored the trajectory of end-of-life symptoms in elderly non-cancer patients receiving care at home or in nursing homes across 59 hospitals and clinics in Japan. All non-cancer patients aged 65 years or older who initiated home or nursing home visits between January 1, 2020, and December 31, 2020, were enrolled in this study. The follow-up period was from 1 January 2020 to 31 December 2021. Patients received routine care, including assessment and treatment, provided by primary care physicians experienced in home-based palliative care. The doctor or nurse in charge at each medical institution registers new patients on the day the home visit starts and records the survey items on the day the home visit starts (data at start), 3, 6, 9 and 12 months after the start, when home care is discontinued or when the patient dies. Participants : Eligibility criteria were: 1) patients aged 65 years and over; 2) patients receiving care at home or in a nursing home. Exclusion criteria were: 1) patients with advanced cancer and 2) patients/families who refused to participate in the study. Age, sex, place of death, major diseases, reason for death, target symptoms for sedation, medications for symptoms, medications used for sedation, sedation methods, duration of sedation, and duration of home care were also measured.

We defined sedation based on previous studies to standardise participants’ understanding [7]. Intermittent and continuous deep sedations were considered palliative sedation. Intermittent sedation was defined as the intermittent administration of sedatives (benzodiazepines, propofol, and barbiturates) to relieve treatment-resistant symptoms. Continuous deep sedation, framed as sedatives (benzodiazepines, propofol, and barbiturates), was administered continuously or regularly to relieve treatment-resistant symptoms and keep the patient in a coma until death. Patients receiving a midazolam dose < 10 mg/day and those taking insomnia medications were excluded in order to focus solely on sedation for symptom relief. The decision to administer sedation was left to the discretion of the treating physician. Classification into the sedation group was based on responses to a structured item in the survey questionnaire, in which attending physicians were asked to indicate whether palliative sedation had been provided. Patients for whom physicians selected “sedation: yes” were classified as having received palliative sedation and were included in the sedation group accordingly.Fisher’s exact test was used to compare whether there was a difference in symptoms before death between the group that received sedation and the group that did not.All statistical analyses were performed with EZR ver1.61 (Saitama Medical Center, Jichi Medical University, Saitama, Japan), which is a graphical user interface for R (The R Foundation for Statistical Computing, Vienna, Austria). More precisely, it is a modified version of R commander designed to add statistical functions frequently used in biostatistics [8].

## Results

**Fig. 1:**
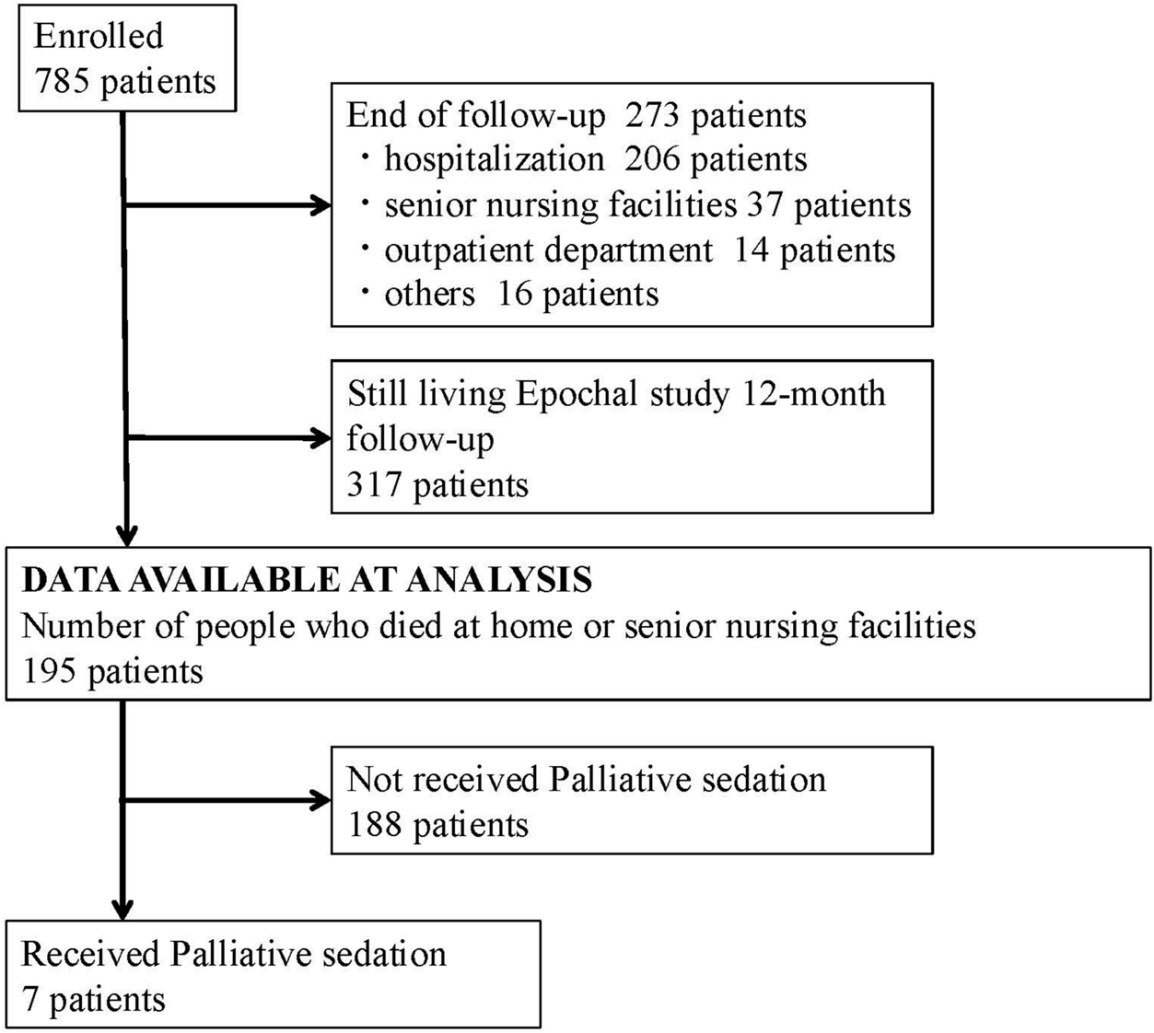
Participant flow

A total of 785 patients were included in the Epochal-J study. Figure 1 shows the patients’ flow diagram. During the one-year follow-up, 195 patients died, with seven patients (3.6%) receiving palliative sedation before death and three of these patients received continuous deep sedation (CDS). The characteristics of the patients who died are shown in Table 1. Palliative sedation was received in 3.8% and 2.7% of patients who died at home and nursing homes, respectively. A summary of patients who received sedation is shown in Table 2.

**Table 1:** Background characteristics of patients with and without palliative sedation

	**With palliative sedation**		**Wituout palliative sedation**		**Total**
	n	%	n	%	(n)
Number of patient	7	3.6	188	96.4	195
Age (years ± SD)	84.3±9.5		86.9±8.7		
Sex					
Male	4	57.1	78	41.5	82
Female	3	42.9	110	58.5	113
Place of death					
Home	6	85.7	152	80.9	158
Nursing Home	1	14.3	36	19.1	37
Others			2	1.1	2
Diseases and conditions requiring home visits					
Dementia	3		56	29.8	59
Heart disease	1		33	17.6	34
Cerebrovascular accident			21	11.2	21
Respiratory disease	2		19	10.1	21
Neurological disease			17	9	17
Kidney disease			11	5.9	11
Musculoskeletal disease			6	3.2	6
Liver disease	1		2	1.1	3
Others			23	12.2	23

**Table 2:** A summary of patients who received sedation in this study

	Age	Sex	Place of death	Major diseases	Reason for death	Target symptoms for sedation	Medications for symptoms	Medicines used for sedation	Sedation methods	Duration of sedation (days)	Duration of home care
1	74	Male	Home	Liver disease, heart failure, kidney failure	Kidney failure	Delirium,dyspnea	Olanzapine	Diazepam (suppository),Midazolam(CSI^b^)	Intermittent sedation,CDS^c^	6	58
2	91	Female	Home	Heart failure, dementia	Senile decay	Delirium	Risperidone	Diazepam (suppository)	Intermittent sedation	3	34
3	80	Male	Home	COPD^a^,cerebrovascular accident	Aspiration pneumonia	Dyspnea	Benzodiazepine, Opioid, Steroid	Midazolam (CSI)	Intermittent sedation,CDS	0.5	118
4	94	Female	Nursing home	Dementia	Senile decay	Delirium	None	Diazepam (suppository)	Intermittent sedation	7	20
5	91	Female	Home	Dementia, COPD	Senile decay	Delirium	None	Chloral hydrate (suppository),Bromazepam (suppository)	Intermittent sedation	5	21
6	90	Male	Home	Dementia	Senile decay	Delirium	Risperidone, Benzodiazepine,Acetaminophen	Benzodiazepines	Intermittent sedation	7	77
7	70	Male	Home	COPD	Pneumonia	Dyspnea	BenzodiazepineOpioid	Midazolam (CSI)	CDS	3	38

^a^*COPD* Chronic obstructive pulmonary disease

^b^*CSI* Continuous subcutaneous injection

^c^*CDS* Continuous deep sedation

Among the target symptoms that received sedation, delirium was present in five patients and dyspnea in three patients (with overlap). Regarding the type of sedation, continuous deep sedation was used for dyspnea, and intermittent sedation was used for delirium. Among the seven patients who received sedation, five (71.4%) experienced delirium within 1 week of death. In contrast, of 177 patients who did not require sedation, 21 (11.2%) experienced delirium. When patients who receivedsedation were compared with those who did not, there was a significant difference in the presence or absence of delirium within 1 week of death (*p*=0.0071).

Among the five patients who received sedation due to delirium, two did not receive medications for delirium. Among patients who did not receive palliative sedation, symptoms rated as “overwhelming” on the IPOS three days before death were as follows (multiple symptoms per patient possible):restlessness (14), poor appetite (9), fatigue (5), dyspnea (2), pain (1), vomiting (1), constipation (1), mouth pain or dryness (1), drowsiness (1), and emotional distress (1).

## Discussion

The most important finding of this study was that palliative sedation was administered to only seven noncancer terminally ill patients (3.6%) in home-based settings and residential care facilities in Japan.

Compared with those for cancer patients, sedation rates for home-based noncancer patients in this study were lower. Sedation rates ranging from 14%−35% have been reported [4, 5]. There are three possible reasons for the low sedation rates observed in this study.

First, there may be differences in the nature and interpretation of symptoms between cancer and non-cancer patients. Previous study reported that non-cancer patients experienced higher intensity levels for certain physical symptoms, including physical pain and shortness of breath [9]. However, in non-cancer patients, such symptoms are often related to chronic diseases where treatment of the underlying condition can contribute to symptom relief. Therefore, even when symptoms are intense, they may not always be perceived as treatment-resistant. In addition, prognostic uncertainty in non-cancer conditions makes it more difficult to determine the terminal phase. This may lead clinicians to hesitate in initiating palliative sedation, as it requires a clear end-of-life determination.

Second, differences in care settings may explain the results. Previous studies in cancer patients have shown that the rate of palliative sedation tends to be lower at home than in hospital. In cancer patients, factors contributing to this difference include limited resources and difficulty in monitoring at home, and the likelihood that hospitalised patients will have more severe symptoms requiring sedation [10, 11]. Although data on palliative sedation rates for non-cancer patients in hospitals remain scarce, it is possible that the same reasons may have contributed to the lower sedation rate in home care observed in this study. For example, the use of subcutaneous midazolam - the most common method of sedation - requires several steps in the home setting: prescription by a doctor, securing the medication, preparing the syringe or infusion pump, and monitoring the patient. Compared with hospitals, where all processes are managed internally, the more complex and resource-limited environment of home care may raise the threshold for administering sedation.

Third, refractory symptoms requiring sedation may not have been adequately assessed by physicians. In this study, patients who did not receive palliative sedation reported symptoms that could potentially warrant sedation - such as dyspnea, pain and psychological distress - within three days of death.It has been reported that general practitioners have lower rates of sedation [10], which might be reflected in these findings. In this study, primary care physicians provided the necessary care. However, in Japan, there is currently no formal training for primary care physicians to provide palliative care at home, and it is left to individual skill. Therefore, training in palliative care, including sedation, is needed. Guidelines from the European Association for Palliative Care concerning palliative sedation are also available for noncancer patients [12]. However, in Japan, there are only guidelines for palliative sedation of patients with cancer [13]. For both palliative care medicine practitioners and general practitioners, there is a need for consensus regarding palliative sedation for noncancer patients.

The second important finding is that, among these cases, three received continuous deep sedation, which was administered to patients presenting with delirium and dyspnea, similar to that of cancer patients [14]. Previous studies on patients with cancer have often administered sedation for symptoms such as delirium, pain, and dyspnea [15]. In home-based care for noncancer patients, pain, delirium, and dyspnea can occur [16].

The strength of this study lies in its focus on noncancer patients receiving home care in Japan. However, this study had several limitations. First, it is unclear whether all physicians understood the differences in palliative sedation. Assessing whether refractory symptoms were appropriately evaluated was difficult in this study. Second, we assessed pain from a doctor’s perspective in this study and could not assess whether the pain was refractory from a patient’s perspective; thus, it was difficult to determine whether palliative sedation was appropriate. Third, this result does not represent the average outcome, as the participants in this study were actively engaged in in-home care. Fourth, the number of study participants was limited, and only a small number of them received palliative sedation, which may reduce the generalizability of the findings. Future studies with larger sample sizes are needed to validate these findings. Fifth, since the method of sedation was left to the discretion of each participant, some methods that are not commonly used were included. Sixth, with regard to physician bias, factors such as individual experience and beliefs, as well as differences in sedation rates according to medical specialty, can be considered. However, as this study used secondary data, it was difficult to account for these biases.Seventh, previous studies have suggested that younger patients tend to receive palliative sedation more frequently [10]. Therefore, differences in age distribution between cancer and non-cancer patient groups may significantly affect sedation rates. While some studies on cancer patients include those younger than 65 years, our study only included patients aged 65 and older. Given the higher frequency of sedation reported among younger patients, this difference in age range may have influenced the observed sedation rates. Given these limitations, future research should be designed with greater methodological rigor and attention to contextual factors, to provide more comprehensive insights and improve the applicability of the findings.

## Conclusion

In this study, palliative sedation was administered to non-cancer patients receiving home-based care, primarily for symptoms such as delirium and dyspnea. However, the overall incidence of sedation remained low at 3.6%.

## Data Availability

The data that support the findings of this study are available on request from the corresponding author. The data are not publicly available due to privacy or ethical restrictions.

## References

[1] Hall S, Petkova H, Tsouros AD, Costantini M, Higginson IJ. Palliative care for older people: better practices. World Health Organization. Denmark: Regional Office for Europe; 2011.

[2] Nicholson C, Davies JM, George R, Smith B, Pace V, Harris L, et al. What are the main palliative care symptoms and concerns of older people with multimorbidity?-A comparative cross-sectional study using routinely collected Phase of Illness, Australia-modified Karnofsky Performance Status and Integrated Palliative Care Outcome Scale data. Ann Palliat Med. 2018;7(Suppl 3):S164–75. 30180731 10.21037/apm.2018.06.07

[3] Heijltjes MT, van Thiel GJMW, Rietjens JAC, van der Heide A, de Graeff A, van Delden JJM. Changing practices in the use of continuous sedation at the end of life: a systematic review of the literature. J Pain Symptom Manage. 2020;60:828–846.e3. 32599152 10.1016/j.jpainsymman.2020.06.019

[4] Mercadante S, Porzio G, Valle A, Fusco F, Aielli F, Costanzo V. Palliative sedation in patients with advanced cancer followed at home: a systematic review. J Pain Symptom Manage. 2011;41:754–60. 21227633 10.1016/j.jpainsymman.2010.07.013

[5] Mercadante S, Porzio G, Valle A, Aielli F, Casuccio A, Home Care-Italy Group. Palliative sedation in patients with advanced cancer followed at home: a prospective study. J Pain Symptom Manage. 2014;47:860–6. 24099896 10.1016/j.jpainsymman.2013.06.019

[6] Hamano J, Shinjo T, Fukumoto K, Kodama M, Kim H, Otomo S, et al. Unresolved palliative care needs of elderly non-cancer patients at home: a multicenter prospective study. J Prim Care Community Health. 2023;14:21501319231221430. 38131120 10.1177/21501319231221431 PMC10748546

[7] Isseki M. Effect of continuous deep sedation on survival in patients with advanced cancer (J-Proval): a propensity score-weighted analysis of a prospective cohort study. Lancet Oncology. 2016;17:115–22. 26610854 10.1016/S1470-2045(15)00401-5

[8] Kanda Y. Investigation of the freely available easy-to-use software ‘EZR’ for medical statistics. Bone Marrow Transplant. 2013;48:452–8. 23208313 10.1038/bmt.2012.244 PMC3590441

[9] Van Lancker A, Van Hecke A, Verhaeghe S, Mattheeuws M, Beeckman D. A comparison of symptoms in older hospitalised cancer and non-cancer patients in need of palliative care: a secondary analysis of two cross-sectional studies. BMC Geriatrics. 2018;18:40. 29402216 10.1186/s12877-018-0721-7 PMC5800068

[10] Tan F, Li N, Wu Y, Zhang C. Palliative sedation determinants: systematic review and meta-analysis in palliative medicine. BMJ Support Palliat Care. 2023;13:e664–675. 10.1136/spcare-2022-004085 PMC10850834 37553203

[11] Calvo-Espinos C, Ruiz de Gaona E, Gonzalez C, Ruiz de Galarreta L, Lopez C. Palliative sedation for cancer patients included in a home care program: a retrospective study. Palliat Support Care. 2015;13:619–24. 24762539 10.1017/S1478951514000200

[12] Surges SM, Brunsch H, Jaspers B, Apostolidis K, Cardone A, Centeno C, et al. Revised European Association for Palliative Care (EAPC) recommended framework on palliative sedation: an international Delphi study. Palliat Med. 2024;38:213–28. 38297460 10.1177/02692163231220225 PMC10865771

[13] Imai K, Morita T, Akechi T, Baba M, Yamaguchi T, Sumi H, et al. The principles of revised clinical guidelines about palliative sedation therapy of the Japanese society for palliative medicine. J Palliat Med. 2020;23:1184–90. 32283043 10.1089/jpm.2019.0626

[14] Caraceni A, Speranza R, Spoldi E, Ambroset CS, Canestrari S, Marinari M, et al. Palliative sedation in terminal cancer patients admitted to hospice or home care programs: does the setting matter? Results from a National Multicenter Observational Study. J Pain Symptom Manage. 2018;56:33–43. 29548893 10.1016/j.jpainsymman.2018.03.008

[15] Arantzamendi M, Belar A, Payne S, Rijpstra M, Preston N, Menten J, et al. Clinical aspects of palliative sedation in prospective studies. A systematic review. J Pain Symptom Manage. 2021;61:831–844.e10. 32961218 10.1016/j.jpainsymman.2020.09.022

[16] Conen K, Guthrie DM, Stevens T, Winemaker S, Seow H. Symptom trajectories of non-cancer patients in the last six months of life: identifying needs in a population-based home care cohort. PLOS One. 2021;16:e0252814. 34129643 10.1371/journal.pone.0252814 PMC8205160

